# Metabolic phenotyping in the mouse model of urinary tract infection shows that 3-hydroxybutyrate in plasma is associated with infection

**DOI:** 10.1371/journal.pone.0186497

**Published:** 2017-10-16

**Authors:** Pei Han, Yong Huang, Yumin Xie, Wu Yang, Yaoyao Wang, Wenying Xiang, Peter J. Hylands, Cristina Legido-Quigley

**Affiliations:** 1 Institute of Pharmaceutical Science, Faculty of Life Sciences & Medicine, King’s College London, London, United Kingdom; 2 Provincial Key Laboratory of Pharmaceutics in Guizhou Province, School of Pharmacy, Guiyang Medical University, Guiyang, Guizhou, China; University of Nebraska Medical Center, UNITED STATES

## Abstract

Urinary tract infection is one of the most common bacterial infections worldwide. Current diagnosis of urinary tract infection chiefly relies on its clinical presentation, urine dipstick tests and urine culture. Small molecules found in bio-fluids related with both infection and recovery would facilitate diagnosis and management of UTI. Mass spectrometry-based fingerprinting of plasma and urine at 3 time points, pre-infection (t = -24h), infection (t = 24h) and post 3-day treatment (t = 112h), were acquired in the following four groups: mice which were healthy, infected but not treated, infected and treated with ciprofloxacin, and infected and treated with ***Relinqing***^®^ granules (n = 6 per group). A metabolomics workflow including multivariate analysis and ROC regression was employed to select metabolic features that correlated with UTI and its treatment. *Circa* 4,000 molecular features were acquired for each sample. The small acid 3-hydroxybutyrate in plasma was found to be differentiated for urinary tract infection, with an area under the curve = 0.97 (95% confidence interval: 0.93–1.00, accuracy = 0.91, sensitivity = 0.92 and specificity = 0.91). The level of 3-hydroxybutyrate in plasma was depleted after infection with a fold change of -22 (*q* < 0.0001). Correlation between plasma 3-hydroxybutyrate and urine bacterial number in all groups and time points was r = -0.753 (*p* < 0.0001). The findings show that 3-hydroxybutyrate is depleted in blood and strongly associated with UTI at both infection and post-treatment stage in a UTI mouse model. Further work is envisaged to assess the clinical potential of blood tests to assist with UTI management.

## Introduction

Urinary tract infection (UTI) is one of the most common bacterial infectious diseases, including a variety of clinical symptoms that range from mild to life-threatening [1, 2]. It is estimated that the incidence of UTI causes near 8 million ambulatory care visits and 1.7 million visits to emergency departments annually in USA. In the UK it accounts for 22% of emergency admissions [3]. UTI affects all age groups with females being at higher risk. As many as 50% females will suffer from urinary tract infections in their life and nearly 25% of them will develop recurrent urinary tract infections [4–6]. UTI often results from both Gram-positive and Gram-negative bacteria, but it can also occur due to a virus or fungus [1, 7]. The most common pathogen is *Escherichia coli* (*E*.*coli*) which accounts for 80–85% of urinary tract infections [8].

To treat UTI is challenging, not only because of the high prevalence and potential morbidity of UTI, but also because of its relatively complicated diagnosis and the prevailing emergence of multidrug-resistant pathogens [1, 9]. Misdiagnosis can cause unnecessary or inappropriate antibiotic treatment which can then result in antibiotic resistance [10]. Delayed diagnosis can lead to failure of appropriate treatment which might cause long-term morbidity, kidney scarring, hypertension and renal damage [11]. Urine culture is regarded as the gold standard for the diagnosis of UTI. The detection of a positive culture result requires at least 18 hours, which can lead to a delayed treatment [11]. Moreover, there is a risk of urine contamination and there is no agreement on the diagnostic cut-off [10, 12]. Clinical presentation poses a great challenge for diagnosis in babies and older people or immunocompromised individuals as it is usually non-specific [13]. Dipstick tests are commonly used in daily practice; however, these tests can have low sensitivity and specificity, hence lacking accuracy [14]. Therefore, a complementary diagnosis in bio-fluid that reflects the host’s response to pathogens in the urinary tract would be valuable for better management of UTI [2].

Discovery studies aim to find markers or indicators that can be objectively measured and used to diagnose the presence or progress of disease [15]. To this end “-omic” technologies provide a hypothesis free platform for the discovery of individual and panels of molecules that associate with clinical outcomes [16]. Metabolomics also called metabolic phenotyping focuses on analysing small metabolites (molecular weight <1 kDa) present in bio-fluids [17]. These metabolites are not only the end products of the pathways of a living system, but also the results of the interaction between genes and the environment [2].

Previous work concerning UTI biomarker discovery focused on metabolites and proteins. Lam CW., *et al*. analysed 88 patients’ urine and reported urine acetic acid alone as a promising UTI biomarker with ^1^H NMR spectroscopy. After this they discovered trimethylamine in urine to correlate with *E*.*coli*-associated UTI with an area under the curve of 0.85 and sensitivity of 0.67 [18, 19]. Yim *et al* suggested that urine neutrophil gelatinase-associated lipocalin (uNGAL), kidney injury molecule-1 (uKIM-1) levels might be useful markers for febrile UTI in children (n = 73 pediatric patients) [20]. Leroy *et al*. conducted a systematic review, involving 18 studies and 1101 patients, and recommended procalcitonin, a propeptide of calcitonin, to be a good predictor to differentiate acute pyelonephritis and lower UTI in children [21]. A summary of studies on UTI biomarker discovery is provided in Table 1.

**Table 1:** Summary of UTI biomarker studies.

Model	Sample	Changed Metabolite	AUC, Sensitivity, Specificity	Ref.
Human	Urine	↑trimethylamine (TMA)	TMA/creatinine AUC = 0.97, sensitivity = 0.91 specificity = 0.95	[18]
Human	Urine	↑acetic acid	Acetic acid/creatinine AUC = 0.97, sensitivity = 0.91 specificity = 0.95	[19]
Human	Urine,	↑ neutrophil gelatinase-associated lipocalin (NGAL), ↑ kidney injury molecule-1(KIM-1)	NGAL/creatinine AUC = 0.90, sensitivity = 0.76 specificity = 0.90 KIM-1/creatinine AUC = 0.66, sensitivity = 0.45 specificity = 0.82	[20]
Rat	Urine	↑neutrophil gelatinase-associated lipocalin (NGAL)	n/a	[23]
Dog	Urine	↑neutrophil gelatinase-associated lipocalin (NGAL)	sensitivity = 0.76 specificity = 0.69	[24]
Human	Urine	↑endothelin-1 (ET-1)	n/a	[25]
Human with UTI recurrence	Urine	↓urinary nerve growth factor (uNGF)	n/a	[26]
Human	Urine	↑neutrophil gelatinase–associated lipocalin (NGAL)	AUC = 0.97, sensitivity = 0.98 specificity = 1.00	[27]
Rat	Urine	↑neutrophil gelatinase-associated lipocalin (NGAL), ↑ kidney injury molecule-1(KIM-1)	NGAL/creatinine (2 days after infection) AUC = 1.00, sensitivity = 1.00 specificity = 1.00 NGAL/creatinine (7 days after infection) AUC = 0.87 sensitivity = 0.80 specificity = 0.80 KIM-1/creatinine (2 days after infection) AUC = 0.87 sensitivity = 0.80 specificity = 0.80	[28]
Human	Serum	↑procalcitonin	n/a(meta-analysis)	[21]
Human	Plasma	↑ soluble urokinase plasminogen activator receptor (suPAR),	n/a	[29]
Human	Urine	↑heparin-binding protein (HBP)	AUC = 0.95, sensitivity = 0.89 specificity = 0.90	[30]
Human	Urine	↑xanthine oxidase (XO) ↑myeloperoxidase (MPO)	XO sensitivity = 1.00 specificity = 1.00 MPO sensitivity = 0.87 specificity = 1.00	[31]
Human	Urine	↑human α-defensin 5 (HD5) ↑human neutrophil peptides (HNP) 1–3	HD5 AUC = 0.86, sensitivity = 1.00 specificity = 0.65 HNP1-3 AUC = 0.88 sensitivity = 0.97 specificity = 0.65	[32]

The main purpose of this study is to scan for metabolites associated to a UTI model, to study the systemic effect caused by bacteria and ideally to be able to monitor the recovery process. With this model, we investigated both plasma and urine longitudinal metabolic fingerprints from a pre-infection to a post-treatment stage. Infected mice were sub-grouped and given different treatments, namely saline, ciprofloxacin, and ***Relinqing***^®^ granules. ***Relinqing***^®^ granules are an SFDA approved drug based solely on *Polygonum capitatum* Buch.-Ham.exD.Don, a herbal medicine long used in south-western China for the treatment of urinary tract infection [22].

## Materials and methods

### Samples and reagents

#### Bacterial strains and culture medium

*Escherichia coli* ATCC25922 bacteria were purchased from the National Centre for Medical Culture Collection (China), Mueller-Hinton Agar culture medium (batch number: 20140924–00), Nutrient Agar culture medium (batch number: 20140304–01) were purchased from Hangzhou Microbial Regent Co., Ltd (China).

#### Chemical reagents

LC-MS grade water, LC-MS grade acetonitrile were purchased from VWR international (UK). LC-MS grade methanol, N,O-bis(trimethylsilyl)trifluoro-acetamide (BSTFA) with 1% trimethylchlorosilane (TMCS), O-methoxyamine-HCL (MOX), succinic-d4 acid were purchased from Sigma-Aldrich (UK). Ciprofloxacin (batch number: 1130026) was purchased from Guangzhou Bai Yun Shan Pharmaceutical General Factory (China). ***Relinqing***^®^ granules (batch number: 141005) was purchased from Guizhou Wei Men Pharmaceutical Company.

### Methods

#### UTI mouse model built and evaluation

**Preparation of bacterial solution**: *E*. *Coli* ATCC25922 was incubated in Mueller-Hinton Agar broth for 24h with shaking at a temperature of 37°C. Turbidimetric tube was used to compare the concentration of culture. Then the *E*. *coli* culture was diluted to appropriate concentration with sterile physiological saline to establish urinary tract infection mouse model.

**Study of inoculated *E*.*coli* suspension concentration**: Twenty-four mice were randomly divided into four groups. After anaesthesia with 10% chloral hydrate, the mouse bladder was voided by gentle squeeze of the abdomen. Then the mice in different groups were inoculated with different concentration of *E*. *coli* suspension (1×10^7^、1×10^8^、1×10^9^ and 5×10^9^ cfu/mL). The inoculation volume was 0.05mL. After inoculation, the mice were deprived of water for 4h to ensure the complete interaction of the *E*.*coli* adhesion with cellular receptors in the bladder. Then they were back to normal water and food consumption. The early morning urine was collected 24h later and diluted by 10-fold (10^−1^, 10^−2^, 10^−3^ respectively). 0.5mL of urine was taken for urine culture. Pouring plate method was used to culture diluted urine and the urine samples were placed in 37°C incubator for culture.

**Study of inoculated *E*.*coli* suspension volume**: A similar procedure was applied. Twenty-four mice were randomly divided into four groups and received the same operation as mentioned above. One group was used as the blank control, while the other three groups received different volume of *E*.*coli* suspension (0.05、0.1、0.15 mL) at the concentration of 1×10^7^ cfu/mL. 5 min later, orbital blood was collected and the blood samples were centrifuged at 15000rpm for 5min. The plasma obtained was diluted by 10 times (10^−1^, 10^−2^, 10^−3^ respectively). 0.5mL of serum samples was taken and pouring plate method was applied for serum culture and then bacterial number was counted.

Another eighteen mice were randomly divided into three groups. The mice in different groups were inoculated with different volume of *E*. *coli* solution (0.05、0.1、0.15 mL) at the concentration of 1×10^7^ cfu/mL. Same method was applied to collect and culture urine sample.

**UTI model built** [33]: Animal studies were approved by the Experiment Animal Centre of Guizhou Medical University and carried out in strict accordance with the guidelines of the National Institutes of Health for the Care and Use of Animals in China. Specific-pathogen-free (SPF) KM female mice (20±2g, 7–8 weeks) (certificate no. SCXK 2014–0011) were purchased from Changsha Tianqin Biotechnology Co., Ltd (China). Before the experiments, the mice were allowed one week of acclimatization in the animal quarters under air conditioning (22±2°C) with relative humidity of 50–60%.

Animals were anesthetized with 10% chloral hydrate. Prior to the establishment of the model, the mouse bladder was gently squeezed to empty the urine. During infection, 0.05mL of *E*. *coli* suspension delivering 1×10^9^ cfu/mL was instilled into bladder through a polypropylene tube (inner diameter 0.28mm, outer diameter 0.61mm; Smith Medical Company, UK) over a period of 30s [34]. After inoculation, the mice were deprived of water for 4h and then were back to normal water and food consumption. Healthy control mice (mock-infection) were injected with sterile saline (without *E*. *coli*) into the bladder.

The urine samples collected in the morning before and after the infection were used to assess the sterility and the model. All urine samples were diluted by 10 times, 100 times and 1000 times respectively. 0.5mL of diluted urine was used for urine culture. Urine was incubated at 37°C incubator and the number of bacteria number was counted after 24h.

#### Group information and sample collection

6 mock-infected mice were used as control group (CTR). 18 successfully infected mice were randomly divided into three groups (n = 6 per group), the UTI group (UTI): treated with saline; the ciprofloxacin group (CPF): treated with ciprofloxacin and the ***Relinqing***^®^ granules group (RLQ): treated with ***Relinqing***^®^ granules. Two infection treatments were included to ascertain the association of selected features with bacterial count independently of the mode of action of a specific treatment.

Both UTI and CTR groups were administered saline twice a day for three days. CPF and RLQ group were dosed with ciprofloxacin or ***Relinqing***^®^ granules by oral gavage at 0.081g/kg and 1.73g/kg respectively twice a day for three days. In the plasma and urine multivariate and univariate analysis, UTI, CPF, and RLQ groups were assigned as Infected Group.

**Fig 1:**
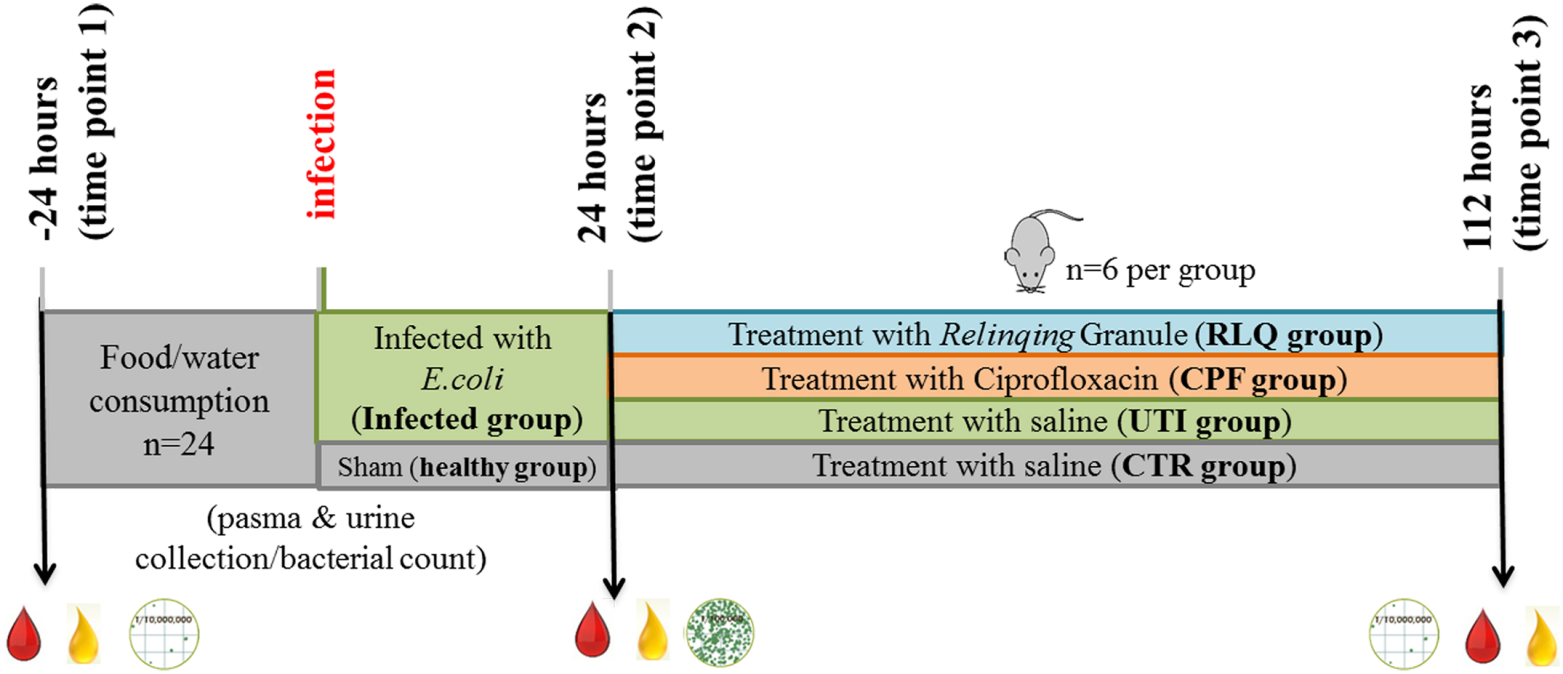
Experimental design for UTI metabolomics study.

Both plasma and 24h-urine samples were collected 24h before infection (time point 1) and 24h after infection (time point 2). Immediately after the collection of time point 2 samples, animals started three-day treatment. 16h after three-day treatment, plasma and urine samples were collected again (time point 3). All the plasma and urine samples (100μL of plasma and 20 μL of urine) were lyophilized and stored in -80°C until use. A figure describing the experimental design can be seen in Fig 1. Table 2 shows the urine bacterial number of mice at different time points.

**Table 2:** Bacterial number in mice urine (cfu/mL).

	CTR Group (n = 6)	UTI Group (n = 6)	CPF Group (n = 6)	RLQ Group (n = 6)
Pre-infection (T1)	0	0	0	0
Infection (T2)	0	(2.87±0.51)×10^5^ ***^a^	(2.72±0.44)×10^5^ ***^a^	(3.24±0.79)×10^5^ ***^a^
Post-treatment (T3)	0***^b^	(2.00±0.69) ×10^5^	0***^b^	(0.26±0.53) ×10^5^ **^b^

Data are expressed as means ± S.D.

cfu: colony forming unit

^a.^ Comparison between CTR and UTI/ CPF/RLQ groups at time point 2 showing the mice were successfully infected, Mann-Whitney test, *** *p* <0.001

^b.^ Comparison between UTI and CTR/CPF/RLQ groups at time point 3 showing the treatments were effective, Mann-Whitney test,

*** *p* <0.001,

** *p*< 0.01

#### Sample preparation for metabolomics analysis

Lyophilized mouse plasma and urine was reconstituted in 100μL/ 20μL of water and vortexed for 2min. Then 300μL/ 60μL of methanol with 20μg/mL of succinic-d4 acid as internal standard added for protein precipitation. Samples were centrifuged for 10 mins at 10,000g at 4°C. 100μL/20μL of supernatant was transferred to a new vial. A pooled sample from all the plasma or urine samples was used as a quality control (QC).

All the samples were dried under N_2_. Then the residue of plasma/urine was added with 50μL/ 20μL of MOX in pyridine (20mg/mL) and heated for 30min at 70°C. Samples were dried down and 100μL/ 30μL of BSTFA (1% TCMS) and 30μL of acetonitrile were added for silylation. The silylation procedure was carried out by incubating the samples at 70°C for 1h. Then samples were pipetted to amber HPLC vials with inserts.

Metabolomics analysis was performed on a Shimadzu GC-2010 Plus gas chromatography system coupled to a GCMS-QP2010 SE single quadruple mass spectrometer (Shimadzu, Kyoto, Japan). Samples were separated on a 30m×0.25mm×0.25μm, BP5MS capillary column. The initial oven temperature was maintained at 60°C for 1 minute, and then raised to 320°C at a rate of 10°C/min, holding for 4minutes. Helium was used as the carrier gas at a flow rate of 40cm/sec. The temperatures for injection, ion source and interface were set at 280°C, 200°C and 320°C respectively. The injection volume was 0.5μL in the split mode with the split ratio of 1:60. Solvent cut time was 5 minutes. Mass data were collected in SCAN mode (m/z 50-600Da) with an event time 0.2s. Electron impact ionization energy was kept at 70eV.

#### Data pre-processing

All GC-MS raw data were converted to mzXML format by the GCMS Postrun Analysis file converting utility (Shimadzu, Kyoto, Japan). The converted data files were then processed using the free “R” package “XCMS” for peak picking and retention time correction. Identification was performed by comparing fragmentation patterns of detected metabolites with the spectra in National Institute of Standards and Technology (NIST) and Shimadzu libraries and then confirmed with the pure standard compound.

#### Statistical analysis

Statistical modelling was accomplished in SIMCA version 14 (MKS Umetrics AB, Sweden). Data were subjected to data modelling, including orthogonal partial least squares-discriminant analysis (OPLS-DA) with corresponding S-plot to mine relevant features. Data were both log-transformed and Pareto-scaled. Selection of discriminating features were based on covariance p[1] and correlation p(corr) values in the S-pot (p[1]>0.10, p(corr)>0.60 and p[1]<-0.05, p(corr)<-0.80). Selected features were measured again in the raw data (area under the peak) and univariate analysis was performed. The receiver operating characteristic (ROC) curve was used to assess the prediction ability of the selected features. All statistical tests including t-test, Mann-Whitney test, Spearman correlation, and Wilcoxon signed ranks test were performed in SPSS (IBM SPSS statistics, version 22). *P* values were adjusted to multiple testing with Benjamini and Hochberg correction when necessary and reported as q-values. A ROC curve was built in “R” with “pROC” package (with a threshold of 0.5). The data treatment workflow for metabolomics discovery is demonstrated in S1 Fig.

## Results

### UTI mouse model built and evaluation

#### Optimization of inoculum concentration and volume

The inoculation concentration and volume of *E*.*coli* suspension were studied. It could be observed that with the increase of concentration, the urine bacterial number was also elevated. However, when the concentration reached 5×10^9^ cfu/mL, the increase of bacterial number was not significant. The results are shown in S1 Table.

Inoculum volume experiments showed a similar pattern where the urine bacterial number increased moderately with the increase of inoculation volume (results shown in S2 Table). Moreover, at inoculum volume of 0.05 ml, plasma cultures of all tested mice were sterile, but when the inoculation volumes were bigger (0.1mL and 0.15mL), plasma cultures of three mice (one with 0.1mL, two with 0.15mL) were positive indicating bacteraemia. Therefore, 0.05mL with concentration of 1×10^9^ cfu/mL was chosen for the UTI mouse model, conditions that were in accordance with Lane *et al* [34].

#### UTI model evaluation

Urine culture was used to assess the status of bacterial growth at different time points. At pre-infection stage (T1), no bacterial colony was observed in the culture medium. At infection stage (T2), the urine culture results were positive for the mice infected with *E*.*coli*. The bacterial numbers can be seen in Table 2.

Plasma creatinine, a biomarker used routinely for kidney function, was also measured before and after infection [35]. Result showed that there was no difference between T1 and T2 indicating no changes in renal function (data not shown).

### Plasma untargeted multivariate analysis

#### Infected group plasma multivariate analysis between T1 and T2

**Fig 2:**
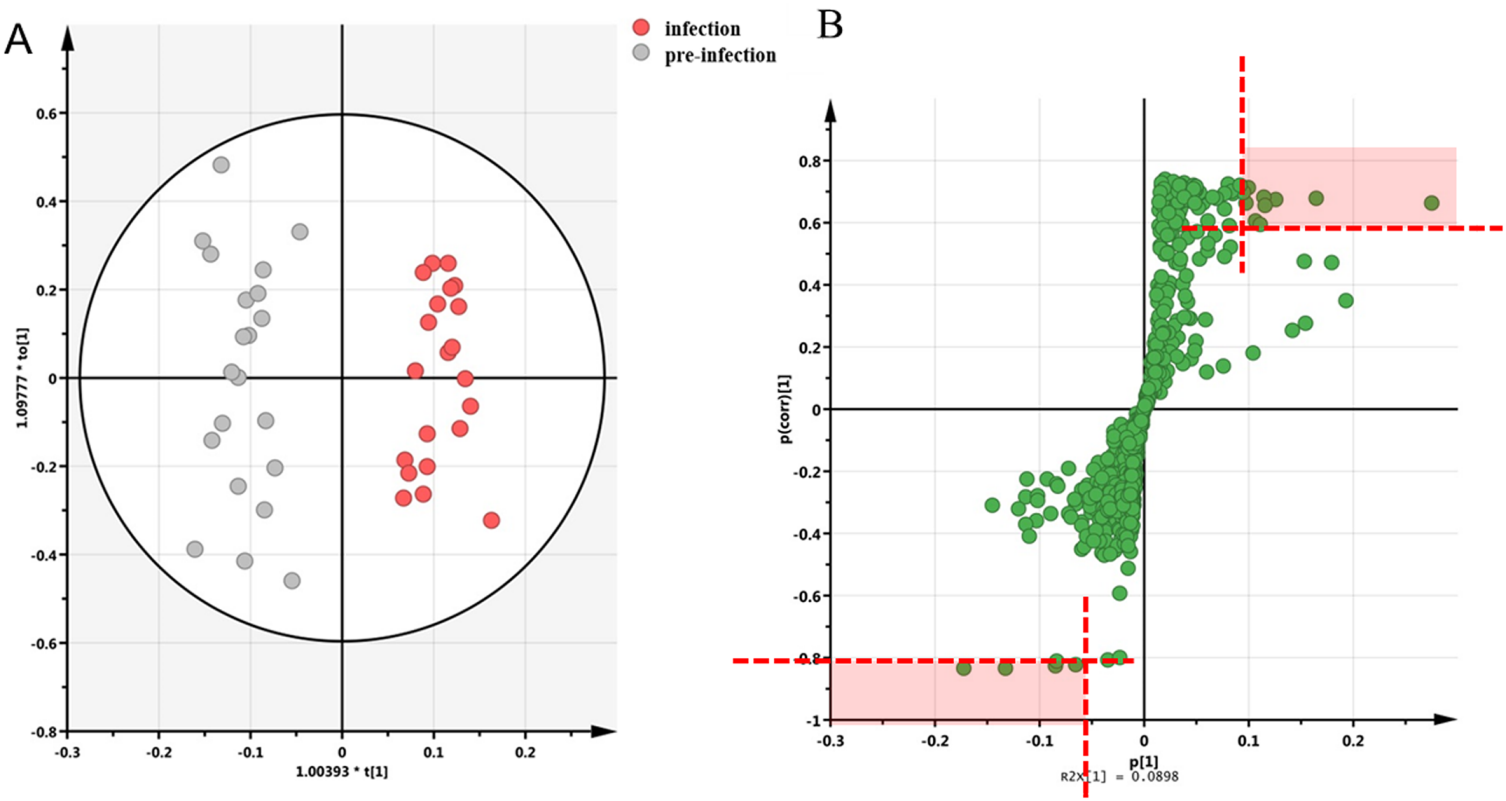
Plasma multivariate analysis showing changes of metabolic fingerprints between pre-infection (T1) and infection (T2) stages in infected group (n = 36 in total). A. OPLS-DA score plot between T1 and T2 with R^2^Y = 0.868, Q^2^ = 0.669 and cross-validated *p*-value = 6.09×10^−7^. R^2^Y showed that 86.8% of group variance was interpreted and Q^2^ represented for the prediction ability of this model with 66.9%. B. S-plot between T1 and T2. Each spot represents for a fragmentation ion.

OPLS-DA model created from GC-MS plasma data revealed a significant variation between pre-infection (T1) and infection (T2) in terms of metabolic profiles (Fig 2A). With this model, four metabolites differentiated pre-infection and infection with p[1]>0.10, p(corr)>0.60 and p[1]<-0.05, p(corr)<-0.80 were selected by the corresponding S-plot (Fig 2B). These four metabolites were identified as lactate, urea, cholesterol and 3-hydroxybutyrate (3-HB).

#### Univariate analysis of selected features

**Metabolite semi-quantification**: The peak areas of four metabolites were measured in the raw data (normalized to internal standard) for univariate analysis. Lactate and cholesterol were excluded after univariate analysis as differences in the raw data were not significant.

**Fig 3:**
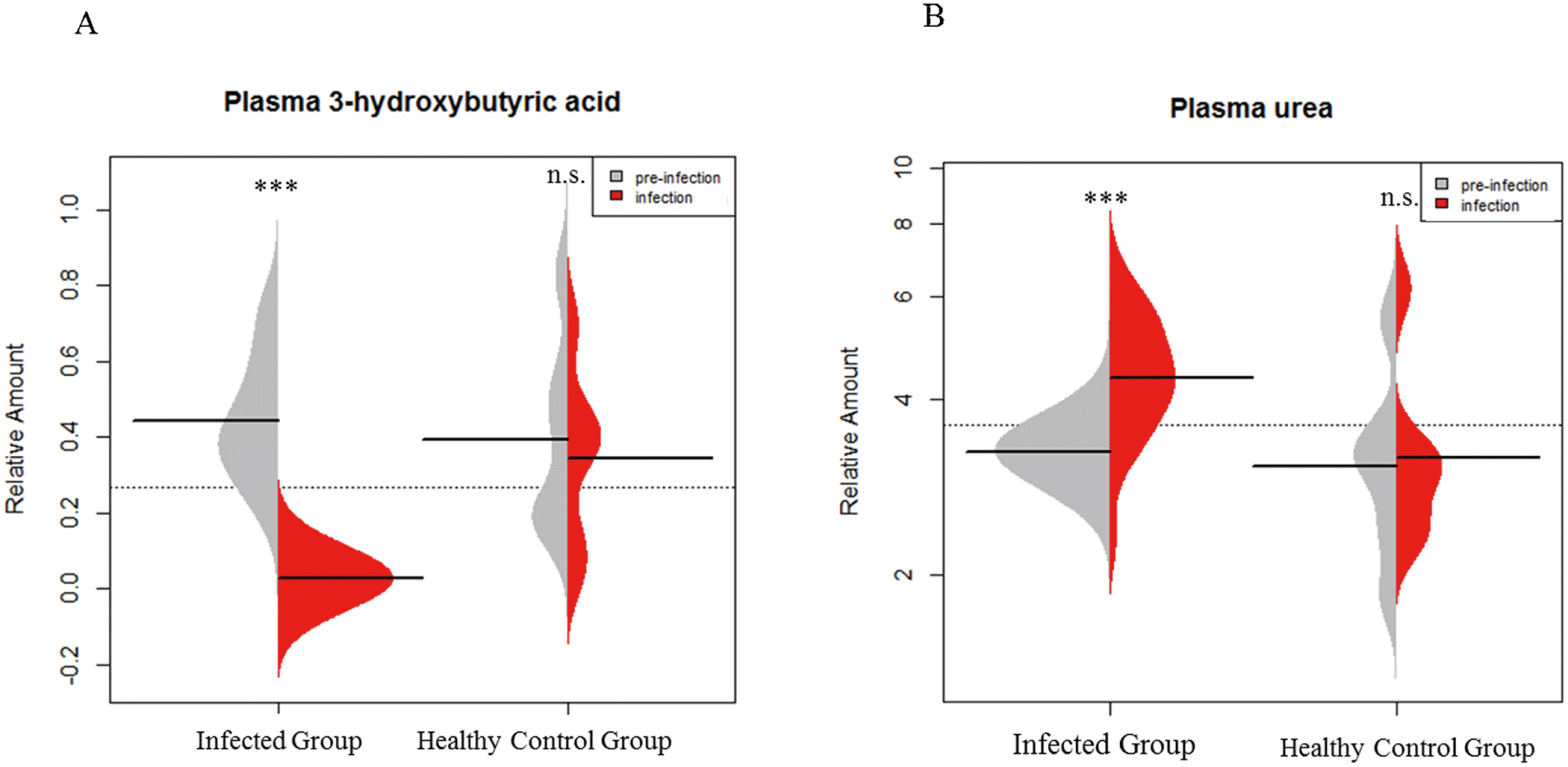
Bean-plots show levels of plasma 3-hydroxybutyric acid and urea at pre-infection (T1) and infection (T2) stages (*** is *q* < 0.001, *q* value is *p* value adjusted by Benjamini and Hochberg correction, n.s.: Not significant). A. Comparison of plasma 3-HB in infected group and healthy control group between T1 and T2 (total n = 36 in infected group, paired t-test, two-tail; total n = 12 in healthy control group, Wilcoxon signed rank test). B. Comparison of plasma urea in infected group and healthy control group between T1 and T2 (total n = 36 in infected group, paired t-test, two-tail; total n = 12 in healthy control group, Wilcoxon signed rank test).

The result of paired t-test (two-tail) with multiple comparison correction indicated that 3-hydroxybutyrate (3-HB) and urea were significantly different after infection in infected group (*q* < 0.001 and *q* < 0.001 respectively) and remained constant in the healthy control group (Fig 3). 3-HB was altered after the infection with 22-fold decrease, whilst urea was elevated with a fold-change of 1.42 (Table 3)

**Table 3:** Summary of semi-quantification data for the selected plasma metabolites in the infected group between T1 and T2.

Metabolite	Fold Change ^a^	*p*-value ^b^	*q*-value ^c^
3-hydroxybutyrate	-22.0	6.09×10^−10^	2.44×10^−9^
urea	1.4	2.5×10^−5^	5×10^−5^
lactate	1.1	6.5×10^−2^	6.5×10^−2^
cholesterol	1.2	5.7×10^−2^	6.5×10^−2^

^a^ The trend of metabolite change was reported as upward (positive value) or downward (negative value)

^b^ *p* value was calculated from paired t-test.

^c^ *q* value is *p* value adjusted by Benjamini and Hochberg correction

**Fig 4:**
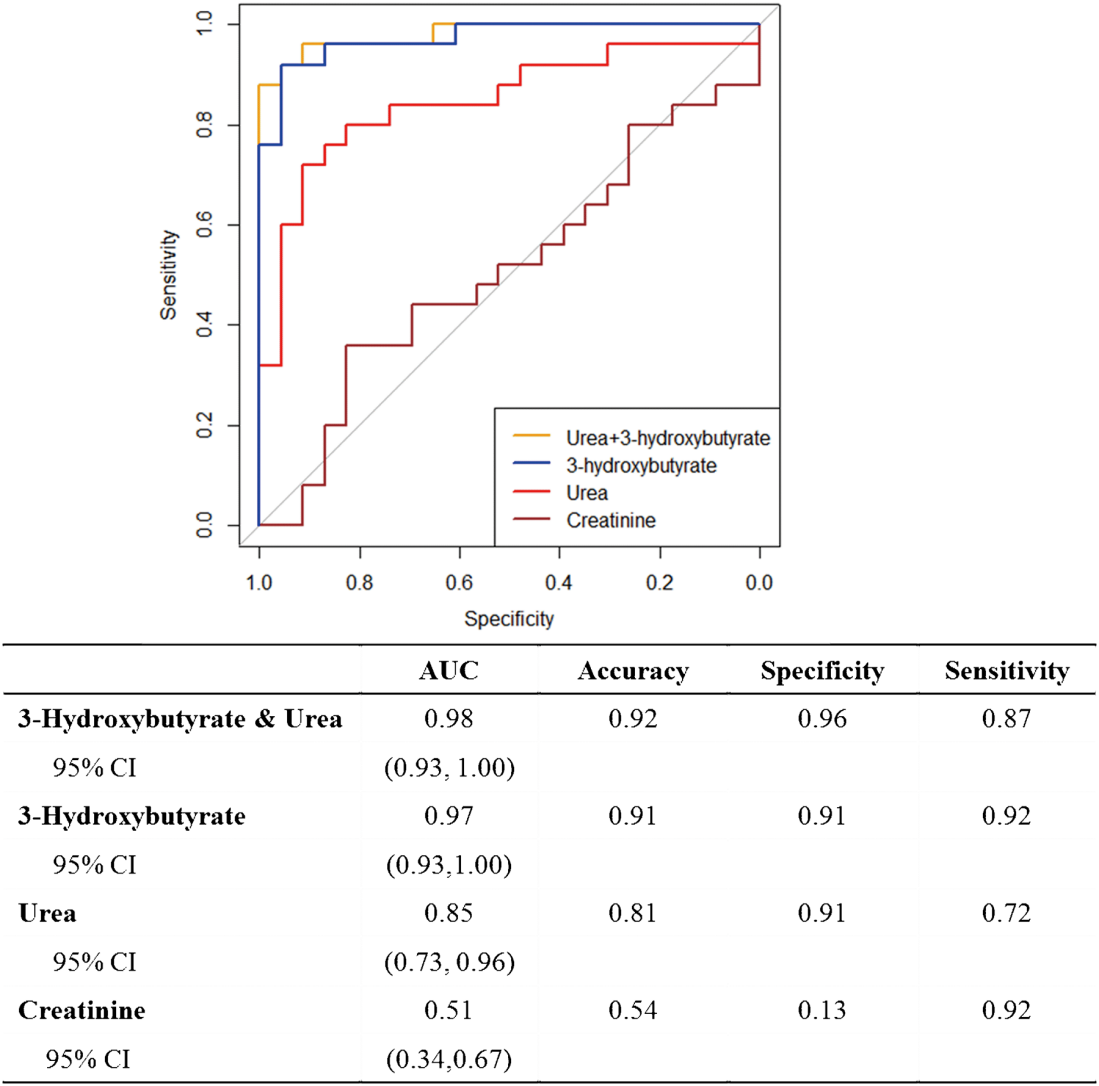
Receiver operating characteristic (ROC) curve prediction ability of UTI based on plasma 3-hydroxybutyrate, urea, creatinine and the combination of 3-hydroxybutyrate and urea. CI: confidence interval.

**ROC curve analysis**: Urea and 3-HB then underwent a receiver operating characteristic (ROC) analysis. Creatinine, the indicator for kidney failure, was also fitted in the analysis to provide a reference. The area under curve (AUC) for 3-HB and urea were 0.97 (95% CI: 0.93–1.00, accuracy = 0.91, sensitivity = 0.92, specificity = 0.91) and 0.85 (95% CI: 0.73–0.96, accuracy = 0.81, sensitivity = 0.72, specificity = 0.91), respectively. The combination of 3-HB and urea produced an AUC of 0.98, but the sensitivity decreased. Therefore, data for 3-HB only were included for further analysis. For comparison, the AUC for creatinine was 0.51 (accuracy = 0.54, sensitivity = 0.92, specificity = 0.13) (Fig 4).

### Urine untargeted multivariate analysis

#### Infected group urine multivariate analysis

Similar data analysis approaches were applied to urine GC-MS data between pre-infection (T1) and infection (T2) stages in infected group. Five features were found to contribute most for group separation. After comparison with the database, they were putatively identified as pyroglutamate, meso-Erythritol and L-(-)-Arabitol, deoxyglucose and D-(+)-Talose.

#### Univariate analysis of selected features

**Fig 5:**
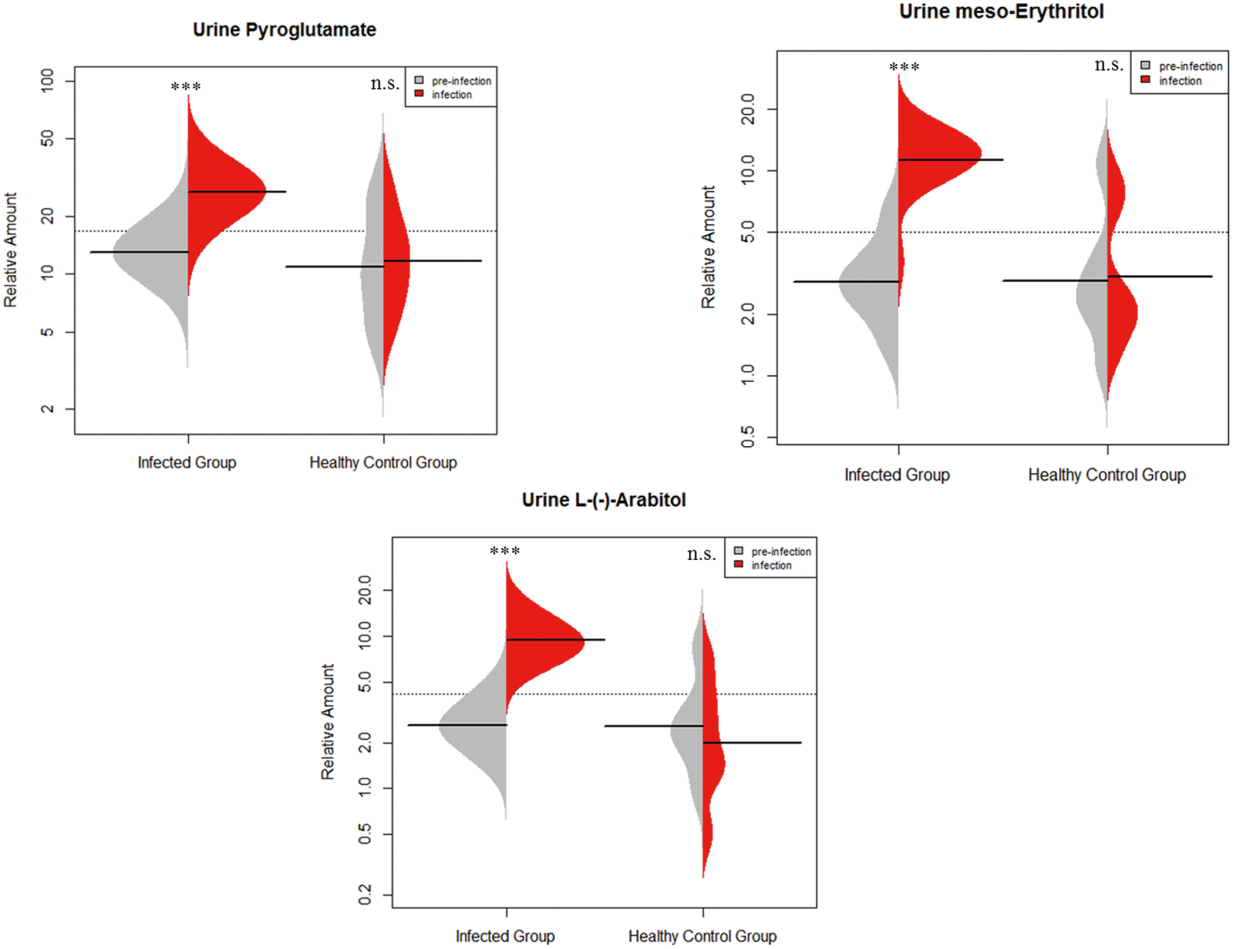
Bean-plots show levels of urine pyroglutamate, meso-Erythritol and L-(-)-Arabitol at pre-infection (T1) and infection (T2) stages (*** is *q* < 0.001, *q* value is *p* value adjusted by Benjamini and Hochberg correction, n.s.: Not significant). Total n = 36 in infected group, paired t-test, two-tail; total n = 12 in healthy control group, Wilcoxon signed rank test.

**Metabolite semi-quantification**: The peak areas of these metabolites were measured in the raw data (normalized to internal standard) for univariate analysis. The result of paired t-test (two-tail) with multiple comparison correction demonstrated that among them pyroglutamate, meso-Erythritol and L-(-)-Arabitol were elevated greatly after infected with *E*.*coli* in infected group (*q* < 0.01) and did not change in the healthy control group (Fig 5). Table 4 presented a summary of semi-quantification data of these metabolites.

**Table 4:** Summary of semi-quantification data for the selected urine metabolites in infected group between T1 and T2.

Metabolite	Fold Change ^a^	*p*-value ^b^	*q*-value ^c^
Pyroglutamate	2.0	6.67×10^−7^	1.11×10^−6^
meso-Erythritol	3.7	1.47×10^−10^	7.35×10^−10^
L-(-)-Arabitol	3.5	3.06×10^−8^	7.65×10^−8^
Deoxyglucose	-1.1	7.02×10^−2^	7.02×10^−2^
D-(+)-Talose	-1.1	6.51×10^−2^	8.13×10^−2^

^a^ The trend of metabolite change was reported as upward (positive value) or downward (negative value)

^b^ *p* value was calculated from paired t-test.

^c^ *q* value is *p* value adjusted by Benjamini and Hochberg correction

**ROC curve analysis**: The predictive abilities of the three metabolites were evaluated by ROC curve as well. All of them gave an area under the curve below 0.80, which was below the desired threshold [36]. When combined these three metabolites, the AUC rose to 0.83 (95% CI: 0.68–0.92) with increased specificity, but no improvement on sensitivity (results shown in S2 Fig).

### Levels of 3-hydroxybutyrate from T1 to T3

#### Plasma levels of 3-hydroxybutyrate

**Fig 6:**
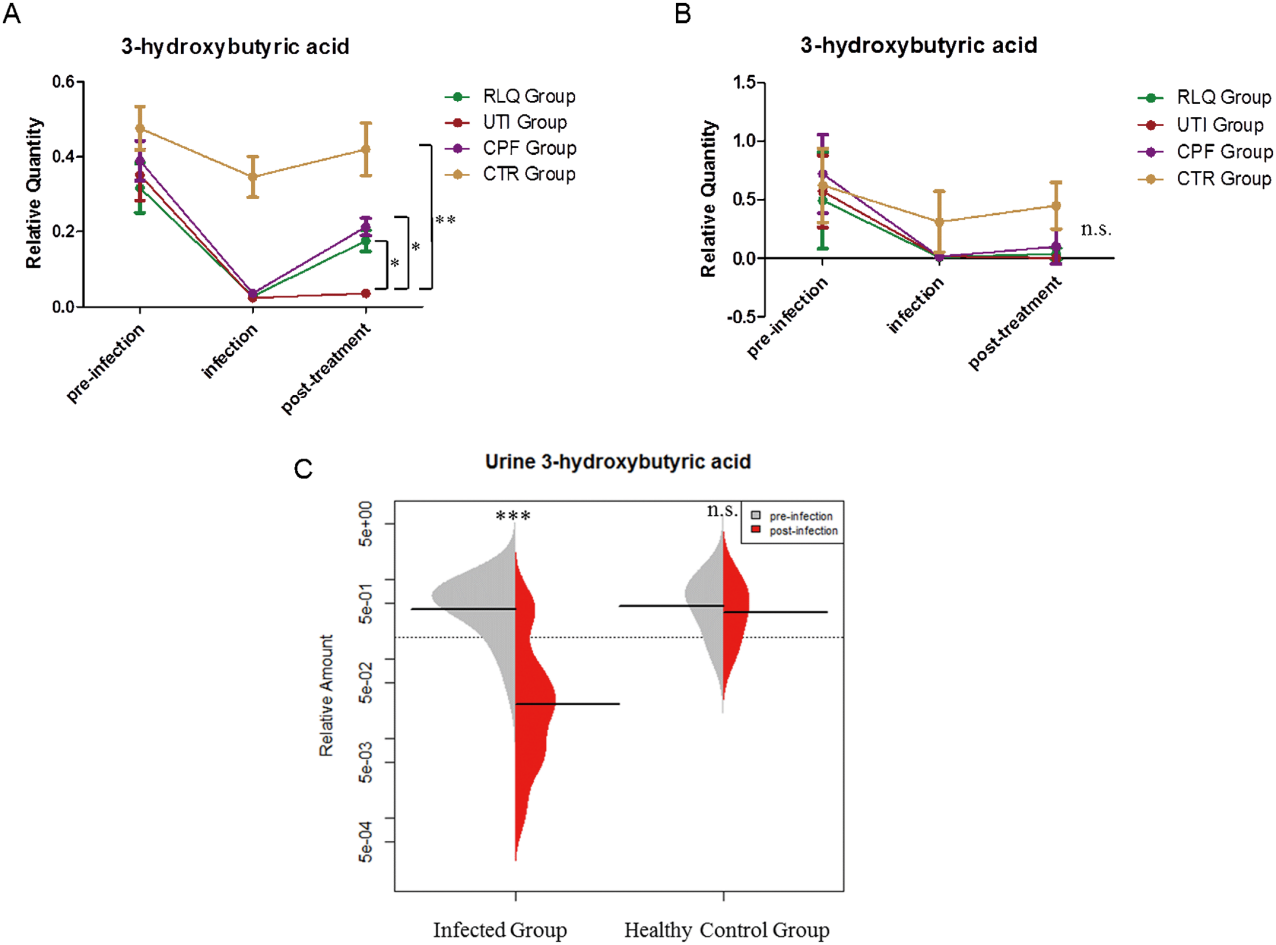
Plasma and urine levels of 3-hydroxybutyrate (* *q* < 0.05, ** *q* < 0.01, *** *q* < 0.001, n.s.: Not significant). A. The change of plasma 3-HB quantity in different groups at different time points (n = 6 per group at each time point). Comparison was made at post-treatment stage (T3) B. The change of urine 3-HB quantity in different groups at different time points (n = 6 per group at each time point). C. Comparison of urinary 3-HB in infected group and healthy control group between T1 and T2 (total n = 36 in infected group, paired t-test, two-tail; total n = 12 in control group, Wilcoxon signed rank test). Results are presented as mean±S.D.

The level of 3-HB in plasma from T1 to T3 was measured. A line graph based on time points was drawn from its semi-quantification data to depict changes at three time points (Fig 6A). The quantity of 3-HB first decreased with infection in all the infected groups while it did not decrease in the control group. With treatments, the level of 3-HB increased after ciprofloxacin or ***Relinqing***^®^ administration (*p* < 0.05 for both CPF and RLQ groups). Table 5 shows the comparison between different groups at different time points. Spearman correlation showed that 3-HB level in plasma strongly correlated with bacterial number, r = -0.754, *p* value < 0.0001 (for RLQ and CPF treatments it was -0.76 and -0.72 respectively).

**Table 5:** Comparison of 3-HB levels in all groups at different time points in plasma and urine.

**Plasma**	**Time Point**	**Comparison**	**Mean (SD)** ^a^	**Mean (SD)**	***p*-Value** ^b^
	**Time Point 1** **(pre-infection)**	UTI vs CTR	UTI = 0.35 (0.17)	CTR = 0.47 (0.25)	0.69
		RLQ vs CTR	RLQ = 0.32 (0.23)	CTR = 0.47 (0.25)	0.78
		CPF vs CTR	CPF = 0.40 (0.13)	CTR = 0.47 (0.25)	0.31
	**Time Point 2** **(infection)**	UTI vs CTR	UTI = 0.023 (0.0096)	CTR = 0.35 (0.21)	0.004
		RLQ vs CTR	RLQ = 0.028 (0.0080)	CTR = 0.35 (0.21)	0.009
		CPF vs CTR	CPF = 0.032 (0.024)	CTR = 0.35 (0.21)	0.010
	**Time Point 3** **(post-infection)**	CTR vs UTI	CTR = 0.42 (0.23)	UTI = 0.030 (0.014)	0.008
		RLQ vs UTI	RLQ = 0.18 (0.069)	UTI = 0.030 (0.014)	0.026
		CPF vs UTI	CPF = 0.21 (0.056)	UTI = 0.030 (0.014)	0.026
**Urine**	**Time Point**	**Comparison**	**Mean (SD)**	**Mean (SD)**	***p*-Value**
	**Time Point 1** **(pre-infection)**	UTI vs CTR	UTI = 0.57 (0.34)	CTR = 0.62 (0.31)	0.19
		RLQ vs CTR	RLQ = 0.49 (0.41)	CTR = 0.62 (0.31)	0.76
		CPF vs CTR	CPF = 0.71 (0.39)	CTR = 0.62 (0.31)	0.47
	**Time Point 2** **(infection)**	UTI vs CTR	URI = 0.017 (0.023) (n = 4) ^c^	CTR = 0.31 (0.28) (n = 5)	0.016
		RLQ vs CTR	RLQ = 0.0097 (0.008) (n = 3)	CTR = 0.31 (0.28) (n = 5)	0.036
		CPF vs CTR	CPF = 0.014 (0.029) (n = 3)	CTR = 0.31 (0.28) (n = 5)	0.036
	**Time Point 3** **(post-infection)**	CTR vs UTI	CTR = 0.44 (0.20) (n = 5)	UTI = 0.019 (0.027) (n = 3)	0.036
		RLQ vs UTI	RLQ = 0.037 (0.049) (n = 5)	UTI = 0.019 (0.027) (n = 3)	0.38
		CPF vs UTI	CPF = 0.10 (0.14) (n = 4)	UTI = 0.019 (0.027) (n = 3)	0.17

^a^ Semi-quantification data obtained by the peak area of 3-HB divided by the peak area of internal standard

^b^ *p*-value obtained from Mann-Whitney test

^c^ number of mice showing the presence of features

SD: standard deviation

#### Urine levels of 3-hydroxybutyrate

The concentration of urine 3-HB also decreased greatly at infection stage in the infected group. In the healthy control group, mice at T1 and T2 showed a similar level of 3-HB (Fig 6C). A line graph based on time points was also depicted in the infected group showing the trend of 3-hydroxybutyrate under different physiological statuses (Fig 6B). Urinary profile also exhibited a lower level of 3-hydroxybutyrate at infection stage (T2) which corroborated the findings of the plasma analysis. In T3 the levels remained depleted in urine, showing that this metabolite was not increased after treatment as it did in plasma during recovery.

### Levels of three urine metabolites during T1 to T3

#### Plasma levels of three urine metabolites

L-(-)-Arabitol and meso-Erythritol were not detectable in plasma, while the amount of pyroglutamate showed no significant difference between pre- and post-infection (data not shown).

#### Urine levels of three urine metabolites

The levels of urine pyroglutamate, meso-Erythritol and L-(-)-Arabitol from pre-infection (T1) to post-treatment (T3) were also measured to investigate any potential of reflecting the infection recovery process. These metabolites, instead of following a clear pattern like plasma 3-hydroxybutyrate, showed changes that were not significant overall. Results are shown in S3 Fig.

The aforementioned findings suggested that 3-hydroxybutyrate might be a candidate metabolite for UTI detection and its management. A pure reference standard of 3-hydroxybutyrate was analysed to confirm the identity of 3-hydroxybutyrate in the samples (S4 Fig).

## Discussion

In our study, we investigated the mouse metabolic response to urinary tract infection with GC-MS-based metabolomics. A significant correlation between 3-HB in plasma and urinary bacterial number/UTI was observed, showing a 22-fold reduction of 3-HB levels in the plasma of *E*.*coli*-infected mice. For the UTI model study, the inoculation concentration and volume were investigated. Plasma culture was used to assess if there was dissemination of bacteria into the blood during infection and plasma creatinine was measured to evaluate kidney function. Before infection, urine samples were collected and cultured to examine if the mice urine was normal. At T1 (healthy) stage no bacterial colony was observed in the culture medium which demonstrated that the urine samples were not contaminated and all the mice were free from bacteria before infection.

### 3-HB association with infection in plasma

To provide an estimate of the prediction ability of selected metabolites, ROC analysis was performed. In ROC curve analysis, sensitivity estimates how good the test is in identifying a disease while specificity evaluates how likely a subject without disease can be ruled out by the test. AUC gives the overall accuracy of the test and can be used to compare different test performances [37]. In plasma, urea as a single measure, 3-HB and 3-HB & urea together provided very high specificity. However, 3-HB generated higher sensitivity of 0.92 to identify mice with UTI compared with urea 0.72 and 3-hydroxybutyrate & urea 0.87.

Urine pyroglutamate, meso-Erythritol and L-(-)-Arabitol combined returned an AUC of 0.83. This suggested a predictive potential of more than 80%, however the sensitivity was only 0.75 and their metabolic variation did not correlate with the recovery process.

After treatment (T3), the levels of 3-HB rebounded in plasma in both CPF and RLQ groups while 3-HB remained unchanged in the UTI group. However, in urine, the level of 3HB didn’t go back. It is because the excretion of 3HB involves renal ultrafiltration, reabsorption and secretion. All of these could make the urine profile show a slower recovery process. By including two different treatments in this experiment, we wanted to increase the reliability of any UTI-related metabolites in plasma. The first treatment, ciprofloxacin, kills bacteria by inhibiting its DNA gyrase [38] and the second, ***Relinqing***^®^ granules, the exact mechanism of action is still unknown. The level of 3-HB increased after the two different treatments, indicating that 3-hydroxybutyrate should be related to infection and the recovery. We observed a slower bacteriostatic effect with the RLQ treatment compared with CPF during the three-day period. This was reflected in both the urine bacterial number and plasma 3-HB levels. The correlation of 3-HB to bacterial number in RLQ and CPF treatments was -0.76 and -0.72 respectively.

### Depletion of 3-hydroxybutyrate

This small acid 3-hydroxybutyrate is one of three ketone bodies together with acetoacetate and acetone. Ketone bodies are generated from β-oxidation of free fatty acids in liver mitochondria (S5 Fig) with three highly regulated enzymatic steps and are the main energy source for tissues when glucose supply is limited [39, 40]. In human, the enzymes involved are different from that in the mouse model, instead of long chain acyl-CoA dehydrogenase (LCAD) playing an essential role in fatty acids oxidation, human mainly rely on very long chain acyl-CoA dehydrogenase (VLCAD) in fatty acids oxidation [41].3-HB decrease with UTI could be related to the suppressed transport of plasma free fatty acid into the liver mitochondria. This mechanism could be initiated by lipopolysaccharide (LPS) which is able to inhibit the enzymes that play a role in the transport of fatty acids.

Lipopolysaccharide, a major component of the outer membrane of *E*.*coli*, is a toxin that is released by *E*.*coli*. It can induce the secretion of pro-inflammatory cytokines [42]. LPS and cytokines can enhance the expression of CD36/fatty acid translocase (FAT) which facilitates the transfer of free fatty acids to the cytosol for re-esterification. They can also reduce the expression of fatty acid-transport proteins (FATP) in the liver, the function of which is to direct free fatty acid to mitochondria for ketogenesis [43, 44]. The increase in CD36/FAT and decrease in FATP result in a lower pool of free fatty acid in the mitochondria, and in turn lead to reduced levels of 3-HB as observed in our data.

**Fig 7:**
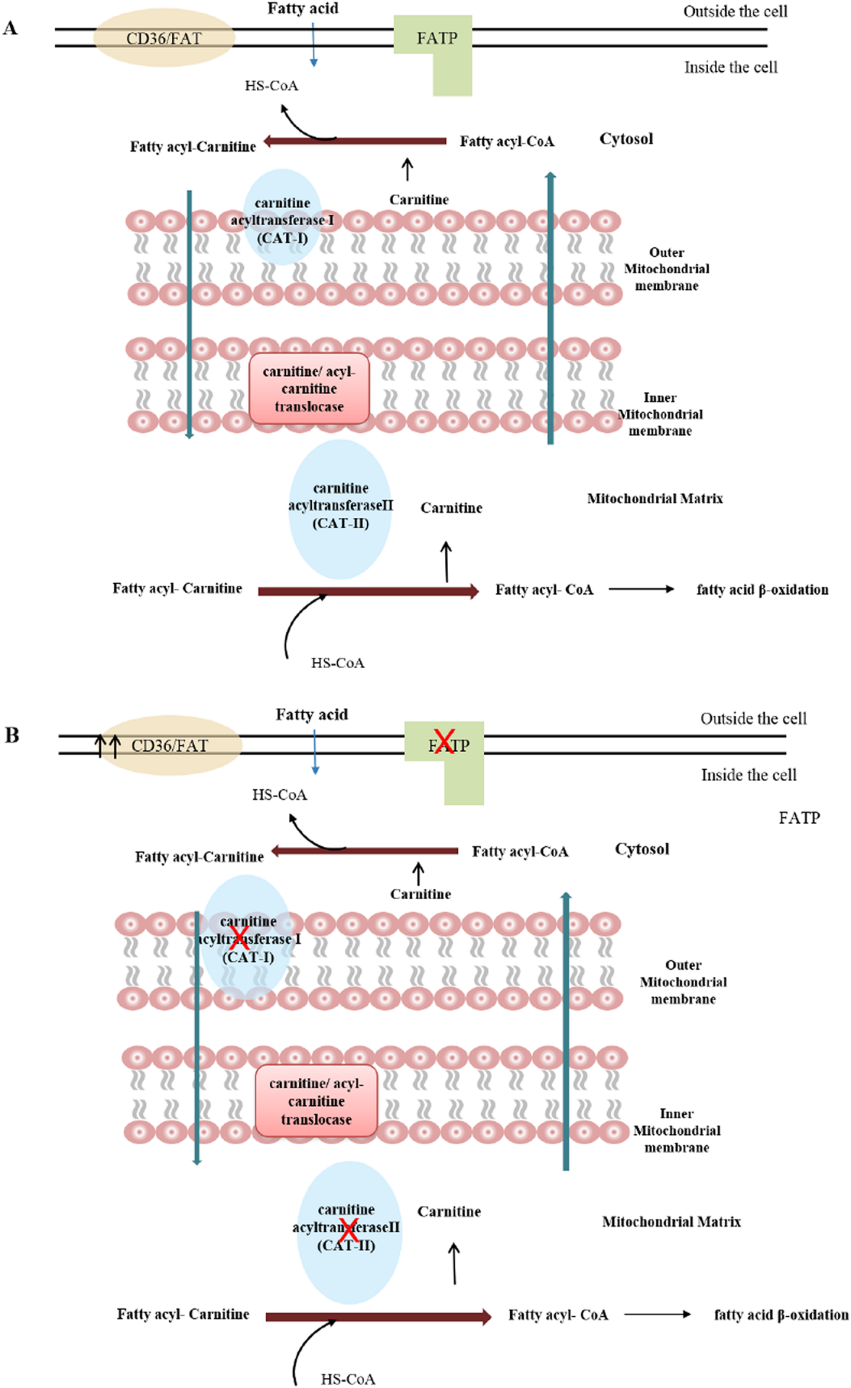
Metabolic pathway of 3-hydroxybutyric acid. A. Transport of fatty acid in control mice B. Hypothesis showing impaired transport of fatty acid in the infected mice.

Moreover, the inner mitochondrial membrane is impermeable for fatty acyl-CoA, it demands a special carrier system to transfer fatty acyl-CoA to the mitochondrial matrix shown in Fig 7A and 7B [45]. In this special carrier system, carnitine acyltransferase I (CAT-I) and carnitine acyltransferase II (CAT- II) are two enzymes that play an important role. LPS has also been reported to suppress the activity of CAT-I and CAT-II [44, 46]. In addition, Neufeld *et al*. and Wannemacher *et al*. observed a decreased concentration of free fatty acid in plasma during infection [47–49].

Plasma metabolic phenotyping is regarded as a collective "snapshot" of changes within the body's metabolism, thus the alteration of plasma metabolites could serve as good indicators of health or disease status [50]. The result presented herein is based on a UTI mouse model, next steps will focus on other bacterial strains as the specificity of 3-HB is not yet proven in these groups.

## Conclusion

Our results indicated that 3-HB in plasma highly correlated to urinary tract infection with a specificity of 0.91 and sensitivity of 0.92. It showed a significant decrease in the *E*.*coli*-infected group and ROC curve revealed that 3-HB had a prediction ability of 0.97 (95% CI: 0.93–1.00). A targeted longitudinal study of plasma 3-HB was also monitored which revealed that 3-HB was able correlate with the recovery process. In addition, the reduced 3-HB was strongly associated with urine bacterial number (r = -0.754, *p* < 0.0001). Further investigations will focus on other bacterial strains in UTI.

## Supporting information

S1 Fig Data treatment workflow illustrates the overall design from untargeted multivariate analysis to semi-targeted univariate analysis and further confirmation study. UTI: mice treated with saline; CPF: mice treated with ciprofloxacin; RLQ: mice treated with ***Relinqing***^®^ **granules**.
(TIF)

S2 Fig Receiver operating characteristic (ROC) curve prediction ability of UTI based on urine pyroglutamate, meso-Erythritol and L-(-)-Arabitol. (TIF)

S3 Fig Urine-targeted analysis of pyroglutamate, meso-Erythritol and L-(-)-Arabitol. A. The change of urine pyroglutamate concentration in different groups at different time points (n = 6 per group at each time point). B. The change of urine meso-Erythritol in different groups at different time points (n = 6 per group at each time point). C. The change of urine L-(-)-Arabitol concentration in different groups at different time points (n = 6 per group at each time point).
(TIF)

S4 Fig A. Chromatogram and mass spectrum of 3-hydroxybutyrate reference standard. B. Chromatogram and mass spectrum of 3-hydroxybutyrate from a plasma sample.
(TIF)

S5 Fig β-oxidation of free fatty acids to 3HB. The enzyme highlighted in yellow is expressed differently in mouse and human.
(TIF)

S1 Table Bacterial number in mice urine at different inoculation concentrations. (DOCX)

S2 Table Bacterial number in mice urine at different inoculation volumes. (DOCX)
