# Supplementary figures and images for: Metabolic phenotyping in the mouse model of urinary tract infection shows that 3-hydroxybutyrate in plasma is associated with infection
Source: PLoS One. 2017 Oct 16;12(10):e0186497. doi: 10.1371/journal.pone.0186497 (PMC5643114; doi:10.1371/journal.pone.0186497)

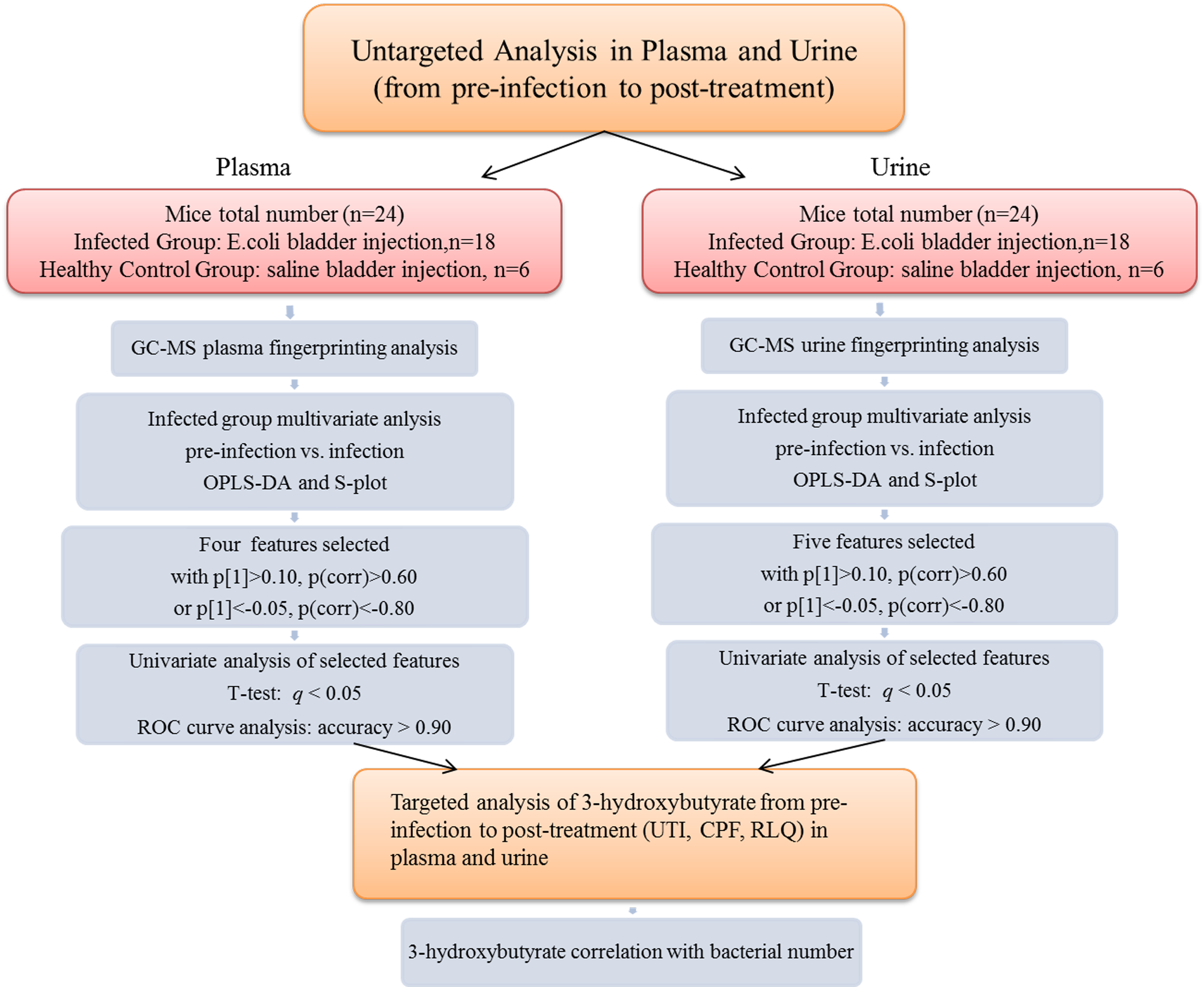

Supplement: S1 Fig — UTI: mice treated with saline; CPF: mice treated with ciprofloxacin; RLQ: mice treated with Relinqing® granules. (TIF) [file pone.0186497.s001.tif]

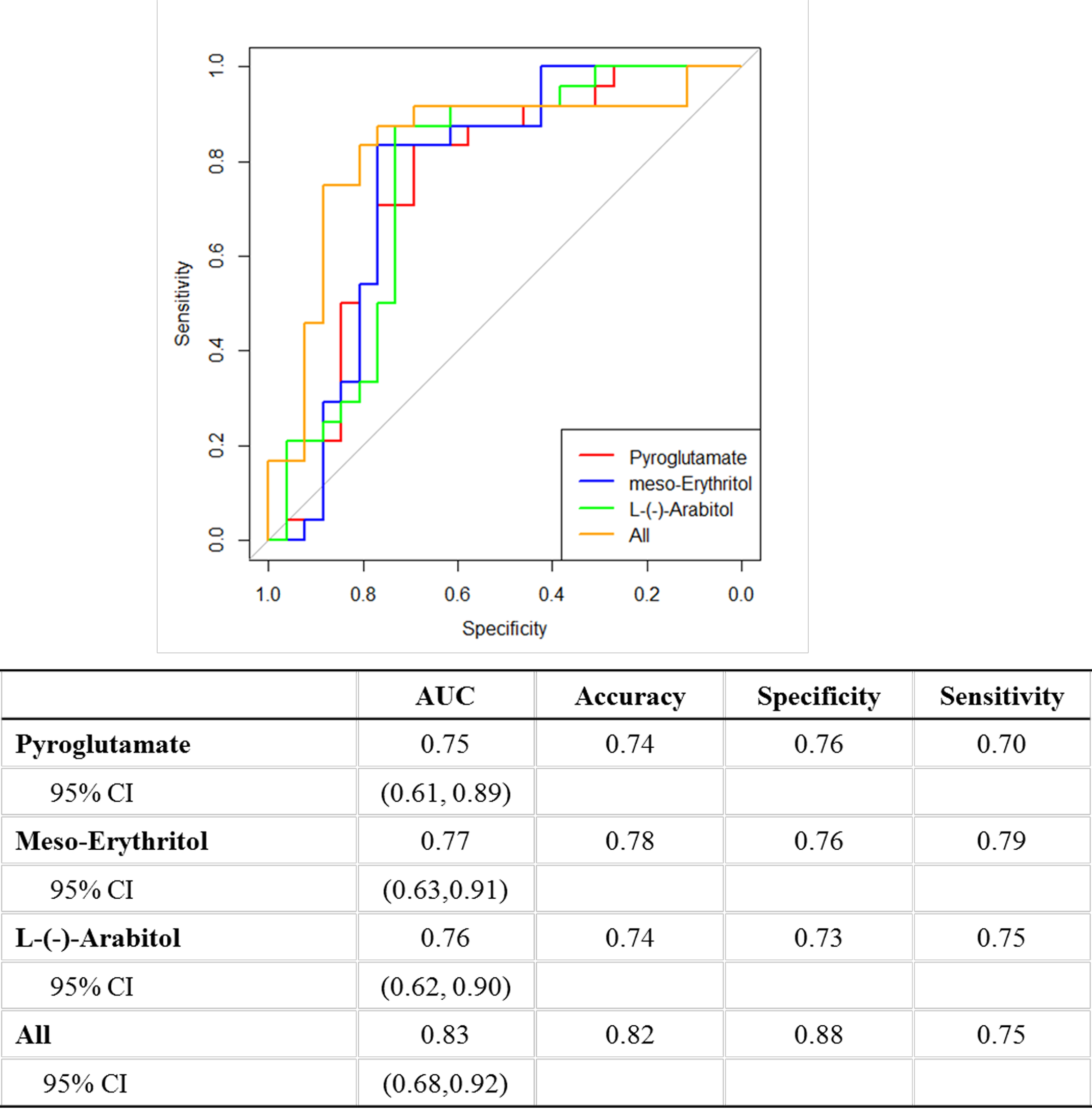

Supplement: S2 Fig — (TIF) [file pone.0186497.s002.tif]

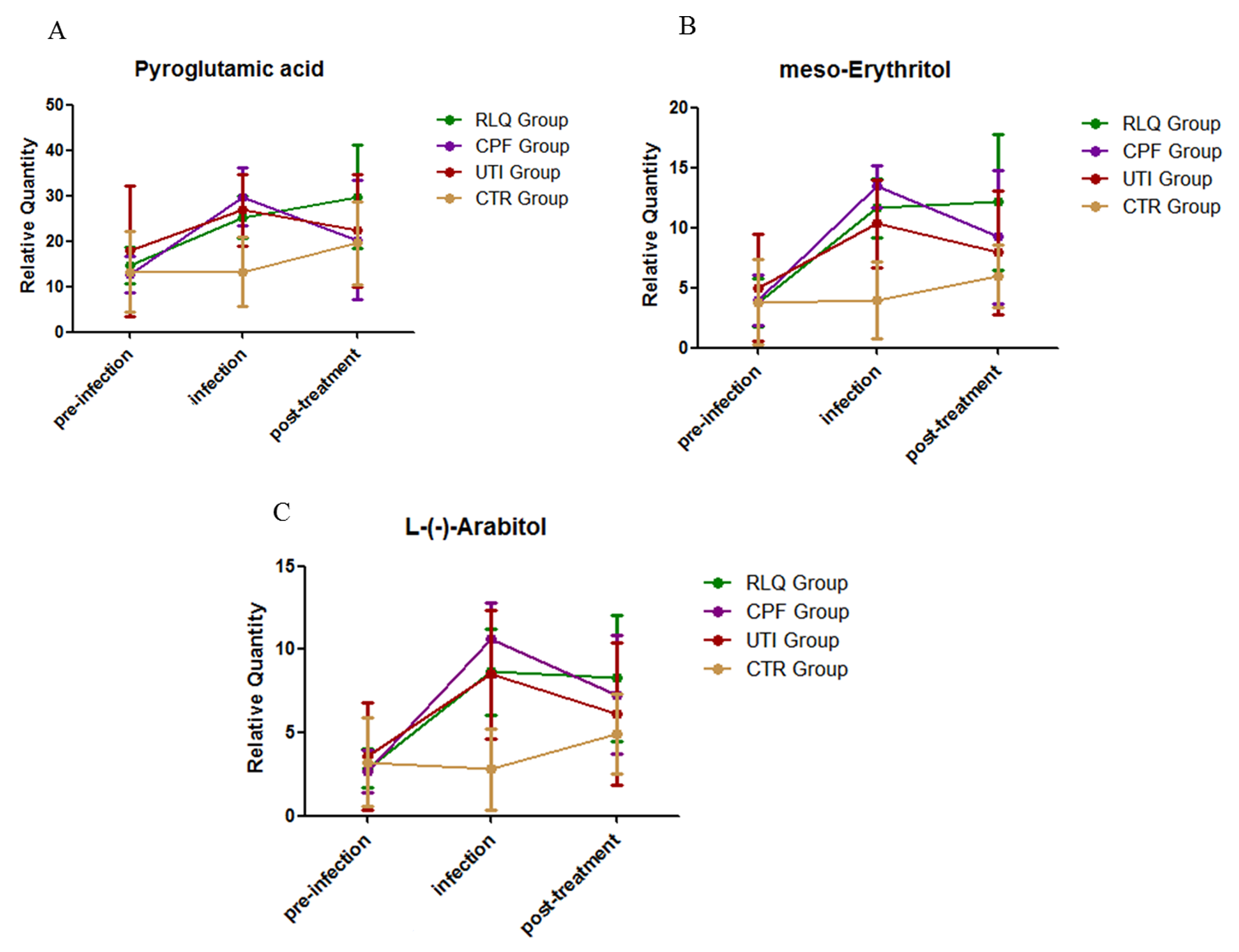

Supplement: S3 Fig — A. The change of urine pyroglutamate concentration in different groups at different time points (n = 6 per group at each time point). B. The change of urine meso-Erythritol in different groups at different time points (n = 6 per group at each time point). C. The change of urine L-(-)-Arabitol concentration in different groups at different time points (n = 6 per group at each time point). (TIF) [file pone.0186497.s003.tif]

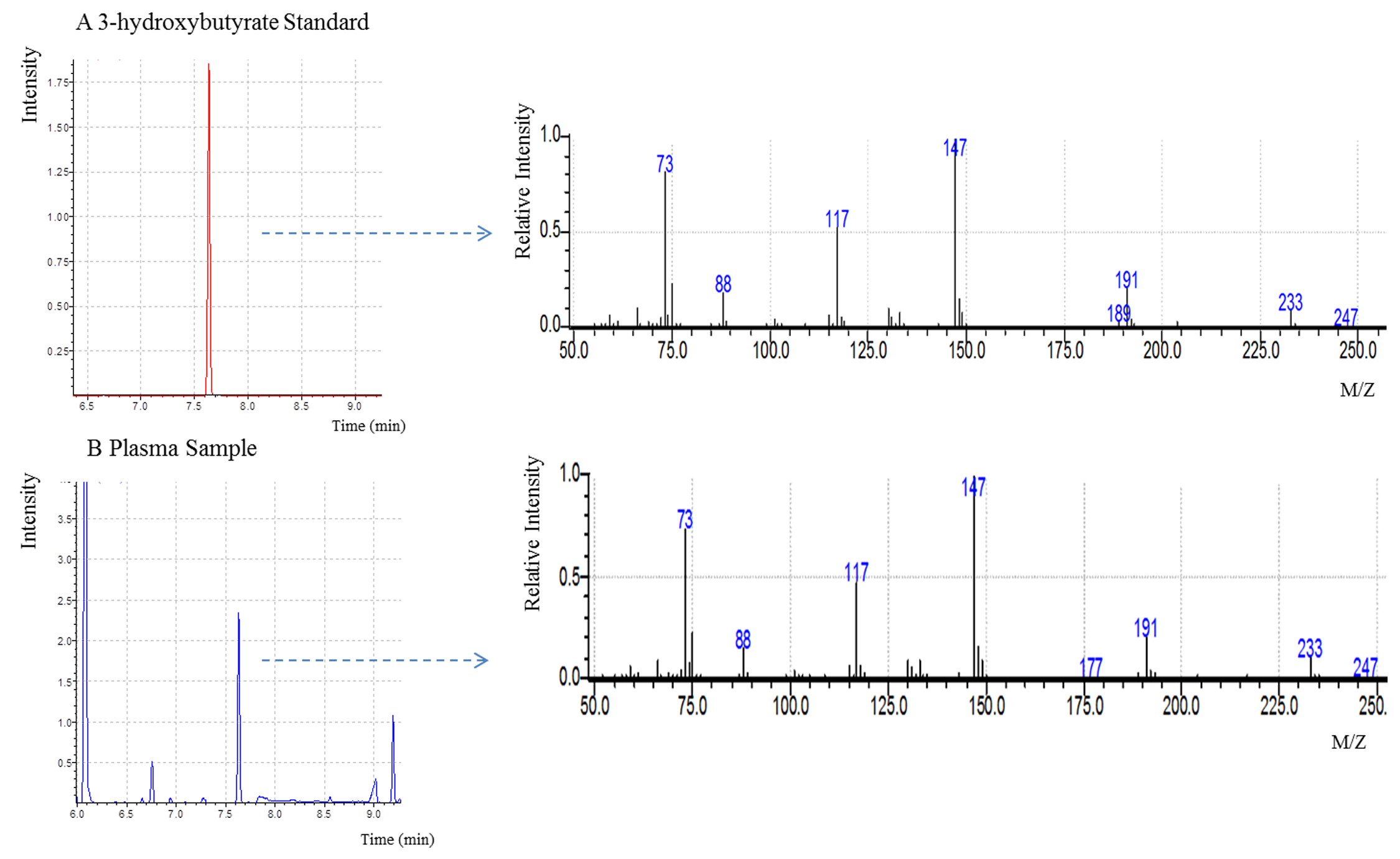

Supplement: S4 Fig — A. Chromatogram and mass spectrum of 3-hydroxybutyrate reference standard. B. Chromatogram and mass spectrum of 3-hydroxybutyrate from a plasma sample. (TIF) [file pone.0186497.s004.tif]

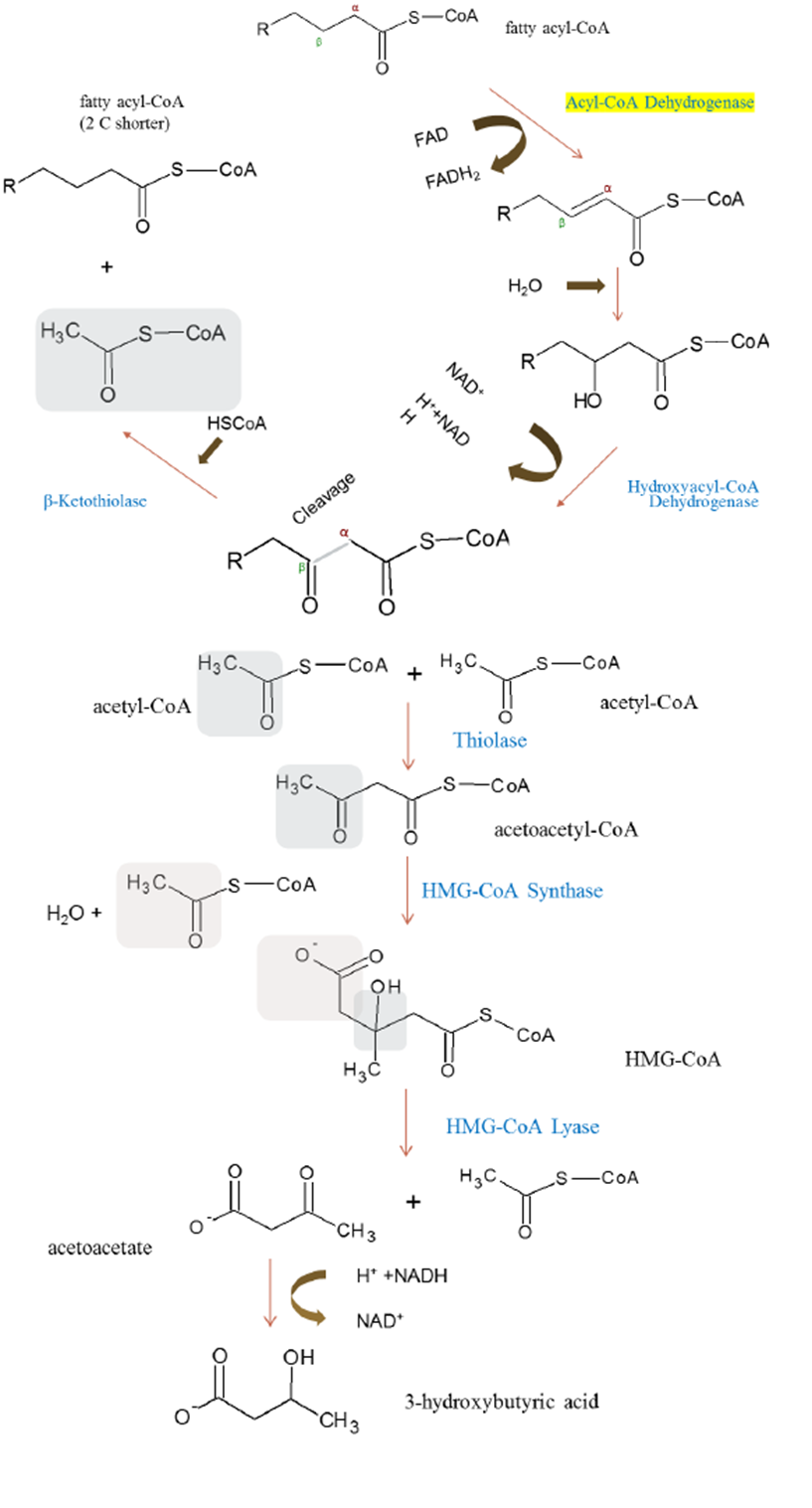

Supplement: S5 Fig — The enzyme highlighted in yellow is expressed differently in mouse and human. (TIF) [file pone.0186497.s005.tif]
