# Supplementary material for: Metabolic phenotyping in the mouse model of urinary tract infection shows that 3-hydroxybutyrate in plasma is associated with infection
Source: PLoS One. 2017 Oct 16;12(10):e0186497. doi: 10.1371/journal.pone.0186497 (PMC5643114; doi:10.1371/journal.pone.0186497)
Supplement: S1 Table — (DOCX) [file pone.0186497.s006.docx]

**S1Table Bacterial Number in Mice Urine at Different Inoculation Concentrations (cfu/mL)**

|  | 1×10^7^ | 1×10^8^ | 1×10^9^ | 5×10^9^ |
| --- | --- | --- | --- | --- |
| Pre-inoculation | 0 | 0 | 0 | 0 |
| Post-inoculation | (0.028±0.0023) ×10^5^ | (0.32±0.030)×10^5***a^ | (2.91±0.12)×10^5**b^ | (3.52±0.23)×10^5n.s.c^ |

Data are expressed as means ± S.D.

cfu: colony forming unit

a. Comparison between 1×10^8^ and1×10^7^, Mann-Whitney test, *** *p* <0.001

b. Comparison between 1×10^9^ and1×10^8^, Mann-Whitney test, ** *p* <0.01

c. Comparison between 5×10^9^ and1×10^9^, Mann-Whitney test, n.s. not significant
