# Supplementary material for: Metabolic phenotyping in the mouse model of urinary tract infection shows that 3-hydroxybutyrate in plasma is associated with infection
Source: PLoS One. 2017 Oct 16;12(10):e0186497. doi: 10.1371/journal.pone.0186497 (PMC5643114; doi:10.1371/journal.pone.0186497)
Supplement: S2 Table — (DOCX) [file pone.0186497.s007.docx]

**S2Table Bacterial Number in Mice Urine at Different Inoculation Volumes (cfu/mL)**

|  | 0.05mL | 0.1mL | 0.15mL |
| --- | --- | --- | --- |
| Pre-inoculation | 0 | 0 | 0 |
| Post-inoculation | (0.29±0.014) ×10^4^ | (0.32±0.015)×10^4*a^ | (0.32±0.02)×10^4n.s.b^ |

Data are expressed as means ± S.D.

cfu: colony forming unit

a. Comparison between 0.1mL and 0.05mL, Mann-Whitney test, * *p* <0.05

b. Comparison between 0.15mL and 0.1mL, Mann-Whitney test, n.s. not significant
